# Natural Antimicrobials Block the Host NF-κB Pathway and Reduce *Enterocytozoon hepatopenaei* Infection Both In Vitro and In Vivo

**DOI:** 10.3390/pharmaceutics15071994

**Published:** 2023-07-20

**Authors:** Iulia Adelina Bunduruș, Igori Balta, Eugenia Butucel, Todd Callaway, Cosmin Alin Popescu, Tiberiu Iancu, Ioan Pet, Lavinia Stef, Nicolae Corcionivoschi

**Affiliations:** 1 Faculty of Bioengineering of Animal Resources, University of Life Sciences King Mihai I from Timisoara, 300645 Timisoara, Romania; iulia_bundurus@animalsci-tm.ro (I.A.B.); balta.igori@usvt.ro (I.B.); eugenia.butucel@afbini.gov.uk (E.B.);; 2 Bacteriology Branch, Veterinary Sciences Division, Agri-Food and Biosciences Institute, Belfast BT4 3SD, UK; 3 Department of Animal and Dairy Science, University of Georgia, Athens, GA 30602, USA; 4 Faculty of Agriculture, University of Life Sciences King Mihai I from Timisoara, 300645 Timisoara, Romania; 5 Faculty of Management and Rural Tourism, University of Life Sciences King Mihai I from Timisoara, 300645 Timisoara, Romania; tiberiuiancu@usvt.ro; 6 Academy of Romanian Scientists, Ilfov Street, No. 3, 050044 Bucharest, Romania

**Keywords:** *Enterocytozoon hepatopenaei*, natural antimicrobials, shrimp, infection, NF-κB pathway

## Abstract

The objective of this work was to investigate, for the first time, the antioxidant effect of a mixture of natural antimicrobials in an *Enterocytozoon hepatopenaei* (EHP) shrimp-gut model of infection and the biological mechanisms involved in their way of action. The study approach included investigations, firstly, in vitro, on shrimp-gut primary (SGP) epithelial cells and in vivo by using EHP-challenged shrimp. Our results show that exposure of EHP spores to 0.1%, 0.5%, 1%, and 2% AuraAqua (Aq) significantly reduced spore activity at all concentrations but was more pronounced after exposure to 0.5% Aq. The Aq was able to reduce EHP infection of SGP cells regardless of cells being pretreated or cocultured during infection with Aq. The survivability of SGP cells infected with EHP spores was significantly increased in both scenarios; however, a more noticeable effect was observed when the infected cells were pre-exposed to Aq. Our data show that infection of SGP cells by EHP activates the host NADPH oxidases and the release of H_2_O_2_ produced. When Aq was used during infection, a significant reduction in H_2_O_2_ was observed concomitant with a significant increase in the levels of CAT and SOD enzymes. Moreover, in the presence of 0.5% Aq, the overproduction of CAT and SOD was correlated with the inactivation of the NF-κB pathway, which, otherwise, as we show, is activated upon EHP infection of SGP cells. In a challenge test, Aq was able to significantly reduce mortality in EHP-infected shrimp and increase the levels of CAT and SOD in the gut tissue. Conclusively, these results show, for the first time, that a mixture of natural antimicrobials (Aq) can reduce the EHP-spore activity, improve the survival rates of primary gut-shrimp epithelial cells and reduce the oxidative damage caused by EHP infection. Moreover, we show that Aq was able to stop the H_2_O_2_ activation of the NF-κB pathway of Crustins, Penaeidins, and the lysozyme, and the CAT and SOD activity both in vitro and in a shrimp challenge test.

## 1. Introduction

*Enterocytozoon hepatopenaei* (EHP), an intracellular shrimp pathogen, is required to transfer germinated spores into the cytoplasm of the host cell in order to complete its life cycle and initiate infection [1]. EHP is responsible for causing hepatopancreatic microsporidiosis (HPM) in shrimp and, once the infection occurs, has a high capacity to spread across other farms and sometimes between countries [2]; as a consequence, shrimp farmers are on constant alert to prevent the emergence of diseases, such as HPM [3]. Hence the identification of interventions, designed to reduce spore germination and subsequent infection, is vital for the industry [4].

The hepatopancreas of shrimp is a critical vital organ involved in the metabolic roles of energy storage, breakdown, and crustacean moulting processes, including nutrient accumulation and lipid as well as carbohydrate metabolism [5]. The EHP infects the hepatopancreatic cell basement of shrimp and triggers the detachment of epithelial cells from the membrane, further utilizing the host’s cell nutrition to reproduce within the cytoplasm of hepatopancreatic tubules, consequently exhausting the cellular energy, and, finally, inducing cell fragmentation and death [6]. The infection also affects crustaceans’ hormonal regulation, immune responses, and signal-transduction pathways and is presumed to increase the sensitivity to other pathogens, such as the *Vibrio* bacteria [6]. The consequences do not necessarily lead to death; however, affected shrimp experience an impaired feed–conversion ratio, which results in retarded growth and enables significant economic disadvantages and losses for aquaculture sectors [7]. One of the possible biochemical mechanisms of shrimp-growth retardation caused by EHP lies behind the activation of the ATP sink [7]. However, recent studies have indicated two other possible mechanisms of induced stunted development [8]. The first is implied in the overstimulation of the juvenile hormones methyl farnesoate, the expression of a vital enzyme involved in juvenile hormone biosynthesis, known as the farnesoic acid O-methyltransferase (FAMet), and decreased expression of the juvenile hormone esterase-like carboxylesterase-1 (JHEC-1), respectively. Another suggested mechanism was associated with the upregulation of ecdysteroid-regulated-like protein (ERP) that impairs the ecdysteroid hormone required to stimulate ecdysis during crustacean moulting [8].

Managing and monitoring EHP infection regularly is required not only for efficient disease control and prevention [9] but also to prevent infection-led changes in the gut microbiomes of shrimp, with further consequences on the nutrition metabolism and immunity [10]. In aquaculture, the prevalent methodology for disease management involves strategically administering chemical substances, such as biocides and antimicrobials, either prophylactically or therapeutically. Those substances could mitigate the effects of disease and maintain the aquatic environment’s health within the farming enclosures. Natural antimicrobials, when used as feed additives, are actually known to improve the growth, survival rate, and meat quality in shrimp, leading to the conclusion that there might be a positive impact on the gut microbiome [11]. Moreover, natural antimicrobial molecules and compounds are known to modulate the activity of the NF-κB pathway [12] which has an important role during infection in farmed shrimp [13].

In the absence of scientific data explaining how organic acids (natural antimicrobials) may influence the virulence of EHP and the downstream impact on host oxidative stress, such research is vital. Mixtures of natural antimicrobials, such as AuraAqua (Aq), were previously shown to reduce oxidative stress, prevent infection, and enhance the immune response in *Nematopsis messor*-infected shrimp epithelial cells [14]. Also, Aq was instrumental in improving the gut health of *Vibrio parahaemolyticus*-infected shrimp gut primary cells (SGP) by stimulating the growth and development of host probiotics such as *Faecalibacterium prausnitzii* [15].

The approach of using antimicrobials in mixtures is justified by their increased efficacy when applied in combinations [16]. Scientific information on the *Enterocytozoon hepatopenaei* mechanisms of infection in shrimp is scarce and regarding the impact of natural antimicrobials is effectively nonexistent. With study, we aimed to improve our knowledge of the EHP infection mechanisms in vitro and in vivo. Moreover, we intended to gather scientific evidence on the biological mechanisms by which natural antimicrobials (Aq) can prevent EHP infection, in vitro, by using gut primary epithelial cells isolated from shrimp or, in vivo, in a *Penaeus vannamei* (*P. vannamei*) EHP challenge infection model.

## 2. Materials and Methods

### 2.1. Isolation and Survival of Spores

The EHP spores were purified as previously described [17] from locally sourced *Penaeus vannamei* shrimp (3–12 g) infected with the microsporidian *Enterocytozoon hepatopenaei* (EHP). The purified EHP spores in distilled water were placed in 96-well plates at 2 × 10^6^ spores/well and incubated with different concentrations of AuraAqua (Aq) for 3 h prior to the Phloxin B extrusion assay, as previously described [17]. The natural antimicrobial mixture, AuraAqua, contains 5% maltodextrin, 1% sodium chloride, 42% citric acid, 18% sodium citrate, 10% silica, 12% malic acid, 9% citrus extract, and 3% olive extract (*w*/*w*). The raw materials were supplied by Bio-Science Nutrition Ireland. Experiments were carried out in triplicates. The most effective concentration was determined by calculating the percentage of spore extrusion. Spores were suspended in 50 μL of 0.1%, 0.5%, 1%, and 2% Aq and incubated for 24–48 h before washing with distilled water. The spore extrusion was examined microscopically.

### 2.2. In Vitro Impact of EHP Infection and AuraAqua Exposure on Shrimp Primary Gut Epithelial Cells (SGP) Survival

The SGP cells were prepared [14] and characterised [18] as previously described. Briefly, the SGP cells were grown in 24 plastic well plates (Analab, Lisburn, UK) in the presence of 0.1% DMSO (Thermo-Fischer, Gloucester, UK) media supplemented with 20% fetal bovine serum (FBS), 100 µg penicillin, 8% shrimp head extract, 6% salt solution, 20 ng epidermal growth factor (Sigma-Aldrich, Gillingham, UK), and 10 U/mL human recombinant interleukin 2 (Sigma-Aldrich, Gillingham, UK). Two experimental approaches were taken: (i) exposure of SGP cells to 0.1%, 0.5%, 1%, and 2% Aq prior to EHP spore infection’ and (ii) inclusion of 0.1%, 0.5%, 1%, and 2% Aq after infection and coculture for 24 additional hours. The SGP cell survival rate was measured by the 3-[4,5-dimethylthiazol-2-yl]-2,5-diphenyl tetrazolium bromide (MTT) assay (Roche, East Sussex, UK; Sigma-Aldrich, Gillingham, UK). Following exposure to EHP, either by using approach (i) or (ii), the plates were incubated under the same conditions (37 °C with 5% CO_2_) and finally washed with 100 μL of the fresh medium prior to measurements. Cell survival was evaluated by adding 10 μL of the MTT reagent (0.5 mg MTT/mL) to each well and incubating for an additional 3 h. This medium was then removed and 100 μL of the solubilization solution was added to dissolve the MTT formazan. The plate was incubated overnight at 37 °C with 5% CO_2_. The absorbance of the MTT purple colour was measured on a multiwell plate reader (FLUOstar Omega, BMG Labtech, UK) using a 570 nm filter. Cell viability was expressed as a percentage of control. To investigate the effect of actin polymerization on H_2_O_2_ release and parasite adhesion, stock solutions of 1 mg/mL of the actin inhibitor cytochalasin D (Sigma-Aldrich, Gillingham, UK) (actin inhibitor) (CytD) were made and diluted to the final concentrations using DMEM culture medium. SGP cells were incubated with 1 μg/mL cytochalasin D for 1 h at 37 °C.

### 2.3. Quantitative PCR

Firstly, for the quantification of EHP adherence to SGP cells, we have carried out a real-time PCR quantification of EHP, as previously described [19]. Infected SGP cells (in 24-well plates) were snap-frozen in liquid nitrogen until use. RNA was isolated using an RNeasy Plus Mini Kit (Qiagen, Manchester, UK). The RNA was reverse transcribed using Transcriptor First Strand cDNA Synthesis Kit (Roche, East Sussex, UK) according to the manufacturer’s protocol. The mRNA levels were determined by quantitative RT-PCR using QuantiNovaSYBR Green PCR Kit (Qiagen, UK) on a LightCycler 96 (Roche, East Sussex, UK). For EHP quantification by real-time PCR, primers F:157 (5′-agtaaactatgccgacaa-3′) and R:157 (5′-ttaagcagcacaatcc-3′), and a TaqMan probe (5-FAM-tcctggtagtgtccttccgt-TAMRA-3′) were used. The real-time PCR protocol included a 20 s initial denaturation at 95 °C followed by 40 cycles of denaturation for 1 s at 95 °C. Annealing and extension took place for 20 s at 60 °C. Secondly, for the mRNA levels of PEN 2, PEN 3, PEN 4, lysozyme, crustin 1, and crustin 2, the primers and the protocol used are as previously described [20] and included in Table 1.

### 2.4. Measurement of H_2_O_2_ Production and SOD and CAT Activity in EHP-Infected SGP Cells in the Presence of AuraAqua

The impact of AuraAqua on superoxide dismutase (SOD) and catalase (CAT) in EHP-infected SGP cells was measured as previously described [21]. Briefly, infected cells, with or without 0.5% Aq treatment, were washed with PBS before the treatment with Trypsin PBS solution. Digested cells were centrifuged for 10 min at 1400× *g*, and the pellet was resuspended in lysis buffer containing protease inhibitors. After 30 min of incubation on ice, the extraction mixture was centrifuged at 12,000× *g* at 4 °C for 30 min and the supernatant was transferred to a fresh tube. SOD activity was determined using a commercially available SOD colourimetric activity kit (Thermo Fisher, Horsham, UK) and CAT by using a catalase activity kit (Abcam, Trumpington, UK, ab83464). The procedures were followed as per manufacturer instructions. NADPH inhibitors including diphenyleneiodonium chloride (DPI, Sigma; 15 µM, 45 min preincubation and wash out) and bovine liver catalase (Sigma-Aldrich, Gillingham, UK; 300 U/mL) were used during the 24 h measuring interval. The gut tissue of challenged shrimp was disrupted by sonication for 60 s (4×) at 4 °C (in ice) in 1% saline solution followed by centrifugation at 2500 rpm at 4 °C for 5 min (Ultrawave DP200-00, Ultrawave Ltd., Cardiff, UK). The supernatant was used to determine the superoxide dismutase (SOD) and catalase (CAT) activity in EHP-challenged shrimp. All experiments were performed in triplicates. For H_2_O_2_ measurement, the cells were routinely grown in 75 cm^2^ tissue-culture flasks (Sigma-Aldrich, Gillingham, UK) in a humidified incubator at 37 °C with 5% CO_2_. The H_2_O_2_ production from infected and uninfected SGP cells, in response to treatment with AuraAqua, was measured using the PeroxiDetect™ Kit (Sigma-Aldrich, Gillingham, UK), following manufacturer guidelines and the previously described procedure [22].

### 2.5. NF-κB Activation Assay

The level of NF-κB p65 activation was measured in the EHP-infected SGP cells after 24 h of infection, after LPS stimulation, or after infection and exposure to 0.5% Aq, as previously described [23]. Briefly, nuclear proteins were extracted using a Nuclear Extraction kit from Abcam (London, UK) and the NF-κB p65 activation in the supernatants was measured using an NF-κB p65 Transcription Factor Assay Kit (Colorimetric) from Abcam according to the manufacturer’s instructions. The level of NF-κB p65 activation was expressed as the ratio of the measured absorbance (OD 450 nm) expressing the quantity of NF-κB p65 activation per 1 mg of total nuclear protein. Colourimetric changes were measured using absorbance at 550 nm and were read using a FLUOstar Omega plate reader (Premier Scientific, Belfast, UK). NF-κB was also measured in the supernatants obtained from the gut tissue of challenged shrimp after disruption by sonication for 60 s (4×) at 4 °C (in ice) in a 1% saline solution followed by centrifugation at 2500 rpm at 4 °C for 5 min.

### 2.6. Western Blotting

For Western blot, the cells were infected, as described above, and lysed by 1× RIPA lysis buffer containing a protease and phosphatase inhibitor cocktail (Thermo Fisher, Horsham, UK). The cell lysates were centrifuged at 12,000× *g* for 10 min at 4 °C and, then, supernatants were collected as previously described [24]. Briefly, prior to separation in 10% SDS polyacrylamide gels, the protein concentration was measured (Thermo Fisher, Horsham, UK) and denatured in 1× Laemmli buffer (Sigma-Aldrich, Gillingham, UK). Proteins were separated on and transferred to 0.45 µm nitrocellulose membrane (Sigma-Aldrich, Gillingham, UK) and blocked in 3% BSA + 0.05% Tween for 30 min. The membranes were blocked with 5% dried milk in Tris-buffered saline and Tween-20 (TBST, 20 mM Tris HCl, 150 mM NaCl, 0.05% Tween-20) for 6 h at room temperature. Subsequently, the membranes were incubated overnight in specific primary antibodies Phospho-NF-κB p65 (Ser536) monoclonal antibody (MA5-15160, 1:500, Thermo Fisher, Horsham, UK) and NF-κB p65 polyclonal antibody (PA5-e16545, 1:200, Thermo Fisher, UK). After extensive washing, the membranes were then incubated with an HRP-conjugated secondary antibody solution for 1 h at room temperature (goat antirabbit IgG (324,300, 1:2000, UK for Phospho- NF-κB p65 and NF-κB p65, Thermo Fisher, Horsham, UK). The membranes were washed three times with TBST; the blots were detected by using enhanced chemiluminescence reagent (ECL) and exposed to photographic films (Kodak, Thermo Fisher, Horsham, UK). Images were collected using the E-Gel Imager from Thermo Fisher, Horsham, UK.

### 2.7. Challenge Tests (Counting Living Larvae)

The impact of Aq on EHP infection was also tested by a challenge test using healthy *P. vannamei* postlarvae, following a procedure previously described and modified for parasitic infection [25]. Our protocol included 120 shrimp postlarvae per replicate, plated in sterile petri dishes and exposed to 2 × 10^6^ spores/well for 24 h. The antimicrobial mixture, Aq, was applied at the time of infection in the concentration of 0.5% and the survival rate was determined 24 h postinfection. A positive and a negative control (±antimicrobial mixture or ±larvae) was also included in the challenge at 0% of the antimicrobial mixture. Measurements of NF-κB, CAT, and SOD were also performed in the gut tissue, as described above. The gut tissue of infected shrimp was also used to quantify by qPCR the mRNA expression levels of AMPs (PEN 2, PEN 3, PEN 4, lysozyme, crustin 1, and crustin 2). The experiment was performed in triplicate.

## 3. Results

### 3.1. The Effect of Aq on EHP Spore Activity and the Survivability of Spore-Infected SGP Cells

**Figure 1:**
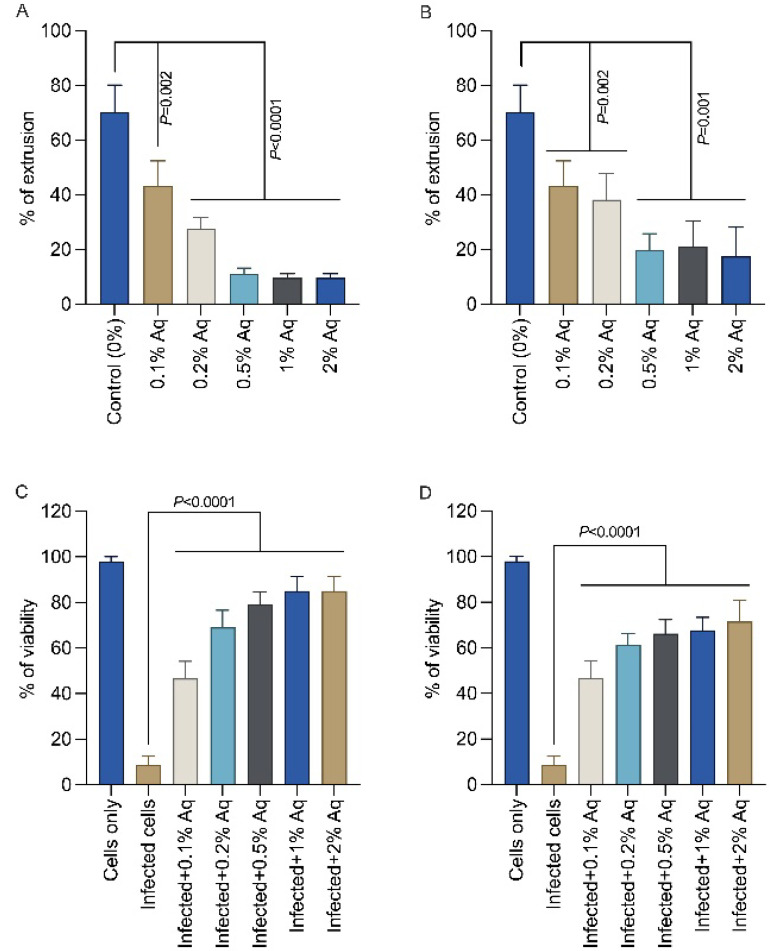
Inhibition of EHP spore activity by Aq after 24 h (Panel (**A**)) and 48 h (Panel (**B**)). The spore activity was evaluated by percentage of extrusion after the addition of Phloxin B. SGP cell viability after exposure to different concentrations of Aq by MTT assay is presented in panels (**C**) (prior infection) and (**D**) (during infection). Cell viability is expressed as a percentage of control cells (assigned as 100%). All experiments were performed in triplicate and the results are represented as means ± standard deviation (SD). Student’s *t* test was performed to assess significance with the *p* values being indicated on graphs.

To determine the impact of Aq on spore activity, EHP spores were exposed to 0.1%, 0.5%, 1%, and 2% Aq, as described in the material and methods. Exposure of EHP spores for either 24 h (Figure 1A) or 48 h (Figure 1B) significantly reduced spore activity at all concentrations, but was more pronounced after exposure to 0.5% Aq. Next, we investigated the impact of Aq during the infection of gut cells and, in doing so, we have infected Aq pre-exposed SGP cells (Figure 1C) or infected cells cocultured with Aq (Figure 1D). The survivability of SGP cells infected with EHP spores was significantly increased in both scenarios; however, a more evident effect was observed when the infected cells were pre-exposed to Aq (Figure 1C). These results suggest that Aq can reduce the EHP spore activity and increase the survivability of EHP-infected SGP cells.

### 3.2. Quantification of EHP in Infected SGP Cells

**Figure 2:**
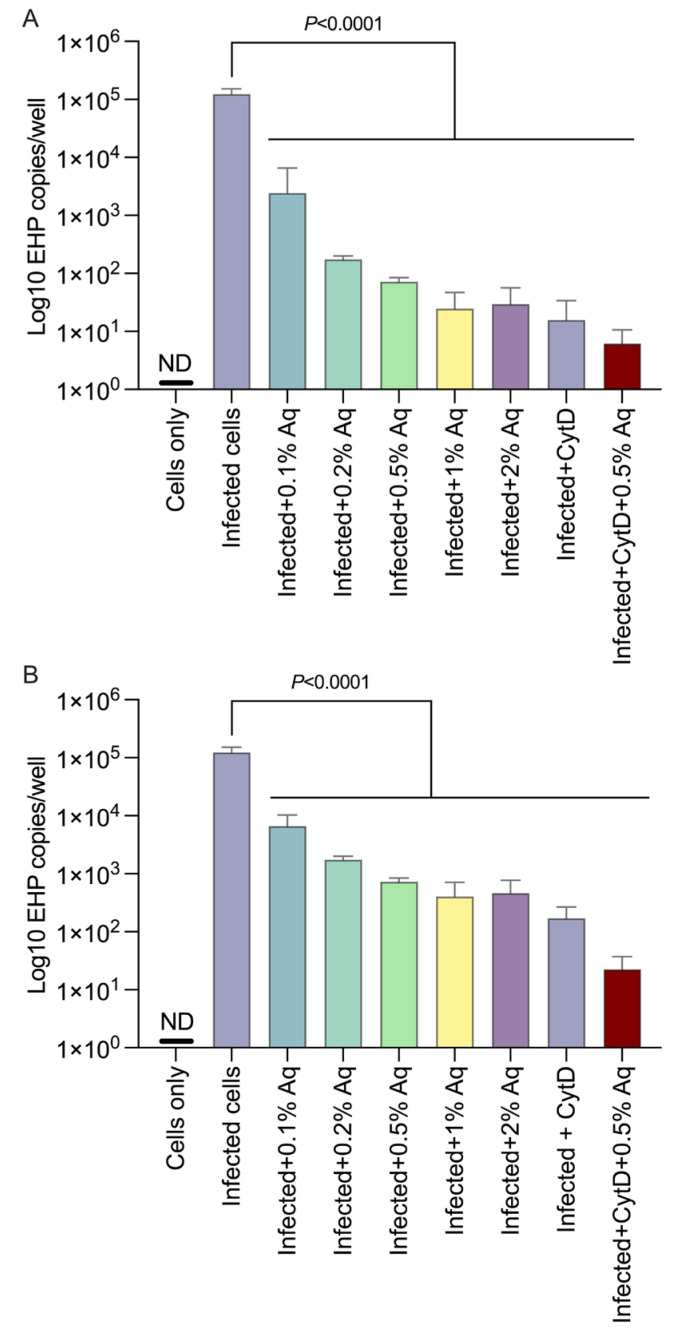
EHP copy number in infected SGP cells. EHP quantification was performed in Aq pretreated and infected SGP cells (panel (**A**)) and in infected SGP cells with Aq present in the culture media throughout infection (panel (**B**)) during the 24 h infection period. All experiments were performed in triplicate and the results are represented as means ± standard deviation (SD). Student’s *t* test was performed to assess significance with the *p* values being indicated on graphs.

To further clarify the antimicrobial effect, we have next investigated the impact of Aq on the EHP spores’ survival by RT-PCR, as described in the material and methods. The number of spores followed a significant decremental pattern (*p* < 0.0001) regardless of Aq being used to pretreat SGP cells prior to infection (Figure 2A) or if Aq was present in the culture media after infection (Figure 2B). However, it is noticeable that the number of EHP copies/infected well was lower if the SGP cells were pretreated with Aq prior to infection. The addition of CytD and of 0.5% Aq, individually or as a dual treatment, suggested that EHP infection requires host action polymerization.

### 3.3. EHP Spore Infection and Oxidative Stress Impact of the Natural Antimicrobial Mixture in SGP Cells

**Figure 3:**
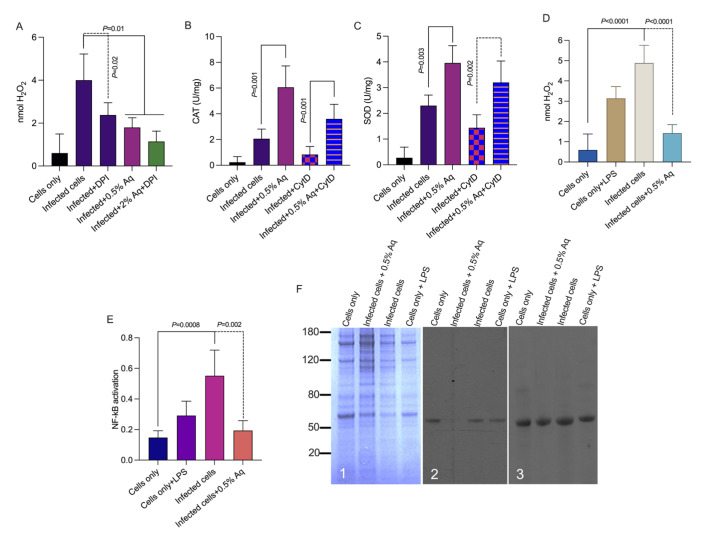
The effect of Aq on EHP-infected SGP cells oxidative stress. Panel (**A**) shows the H_2_O_2_ levels in EHP-infected SGP cells in the presence of Aq or the NADPH inhibitor diphenyleneiodonium chloride (DPI). Panel (**B**) shows the impact on CAT activity and panel (**C**) indicates the SOD activity in infected SGP cells. The impact of Aq on H_2_O_2_ production is shown in panel (**D**) and the role in NF-κB p65 pathway activation is shown in Panel (**E**). Panel (**F**) presents the Coomassie-stained gel of SGP cells lysate (F1), panel F2 shows the anti-p-serine immunoblot of phospho-NF-κB p65 on SGP cells lysate and panel F3 the NF-κB p65 (S536) immunoblot. Data are presented as means (SD) of three independent experiments. *p* values are indicated on graphs.

Our next aim was to investigate if EHP spore infection impacts on H_2_O_2_ release upon EHP spore adherence to SGP cells. In this experiment, we have used 0.5% Aq only as previously identified to have a significant impact on adherence or spore extrusion. As our results show (Figure 3A), there is an increase in the amount of H_2_O_2_ released by the infected cells, which is subsequently reduced in the presence of DPI from ~3.8 nmol to below 2.5 nmol (*p* < 0.05) in infected SGP cells, indicating the activation of host NADPH oxidases upon infection. A similar significant decremental effect was observed in the presence of 0.5% Aq (*p* < 0.05) when the levels of H_2_O_2_ were reduced to below 2 nmol. When both DPI and 0.5% Aq were used, a further decrease was observed but with no difference in significance. Moreover, it became obvious that the reduction in H_2_O_2_ is associated with a significant increase (*p* < 0.05) in catalase production (Figure 3B) and superoxide dismutase (Figure 3C). Both catalase and superoxide dismutase production were lower when CytD was used to block infection of SGP cells, an effect reversed when CytD was used in combination with 0.5% Aq. These results indicate that EHP infection of SGP cells triggers the activation of host NADPH oxidases, an effect which is reversed by the addition of 0.5% Aq. Furthermore, these results indicate that the activation of catalase and superoxidase dismutase activation is not dependent on the physical interaction between EHP spores and the SGP cells. This outcome prompted our next experiment aiming to investigate the involvement of the NF-κB signalling pathway in controlling CAT and SOD production.

This effect will lead to reduced H_2_O_2_ production in infected and Aq-treated SGP cells (Figure 3D). The results presented in Figure 3E clearly indicate that the activation of the NF-κB p65 signalling pathway observed in EHP-infected SGP cells is significantly reduced in the presence of 0.5% Aq and is correlated with loss of P-ser phosphorylation of p65 (Figure 3F). In summary, this data shows that, upon infection and destabilization of the actin cytoskeleton, EHP triggers the host NADPH oxidases to produce H_2_O_2_ responsible for the oxidative activation of the NF-κB pathway via the P-ser phosphorylation of p65. The impact is further cascaded by increasing the production of the CAT and the SOD enzymes in the presence of 0.5% Aq.

### 3.4. Challenge Trial to Determine the In Vivo Effect of Aq

**Figure 4:**
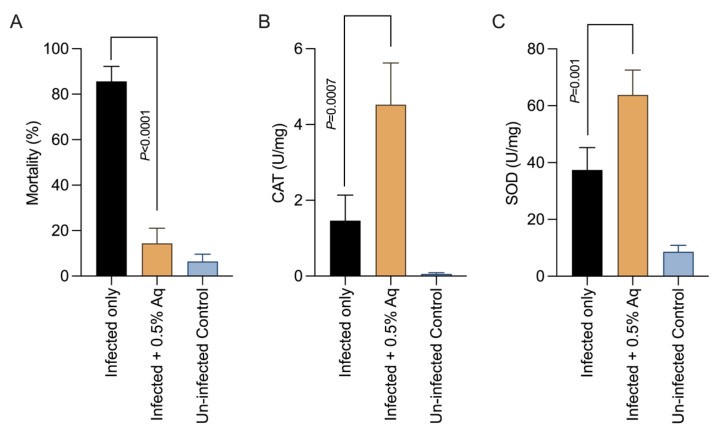
The effect of 0.5% Aq on the antioxidative capacity of *P vannamei*: (**A**) Percentage of mortality, (**B**) CAT activity, and (**C**) SOD activity. Bars and error bars represent the means ± SD. Measurements were performed in triplicates. To express the significance, the *p* values are indicated on graphs.

**Figure 5:**
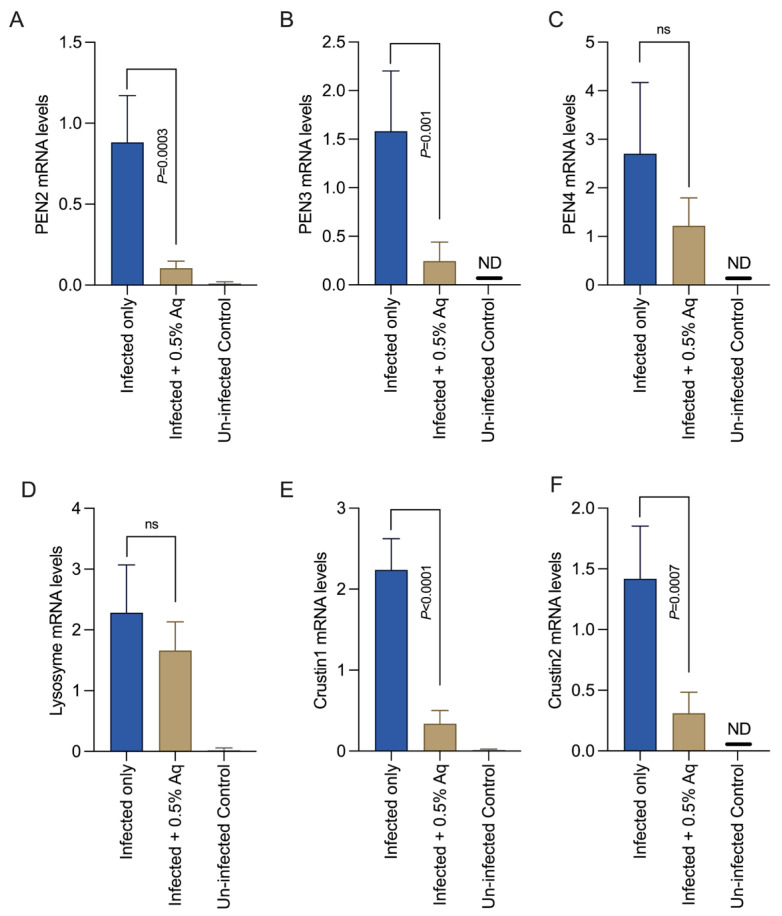
The mRNA expression levels of PEN2 (**A**), PEN3 (**B**), PEN4 (**C**), Lysozyme (**D**), Crustin1 (**E**), and Crustin2 (**F**) in the gut of the infected following exposure or nonexposure to 0.5% Aq of EHP-challenged shrimp, as determined by qPCR. Triplicate independent samples and experiments were conducted.

The results observed so far, in vitro, necessitated the implementation of an in vivo challenge experiment to confirm the observed effect of Aq against EHP infection. *P. vannamei* shrimps infected with EHP with or without Aq treatment were observed for the impact on mortality rates, CAT, and SOD production. The results presented in Figure 4A show that 0.5% Aq reduced to less than 20% of the mortality rates in EHP-infected shrimp at 24 h exposure. Figure 4B indicates that catalase followed had a tendency of increased activity (*p* < 0.0001) in the presence of 0.5% Aq in the infected group. The SOD (Figure 4C) was also significantly increased (*p* = 0.001) in the infected and treated gut tissue. Next, we aimed to determine if the in vitro observed effect on the NF-κB is also mirrored in vivo by measuring the NF-κB pathway-mediated antimicrobial peptides (AMPs) PEN2, PEN4, PEN4, lysozyme, crustin 1, and crustin 2 (Figure 5). As shown in Figure 5, EHP-infected shrimp treated with 0.5% Aq produced mRNA levels, of all these AMPs, significantly reduced (*p* < 0.05) compared to the infected control, apart from PEN4 and lysozyme where the decrease was not significant. Conclusively, these data confirm that Aq can reduce the negative effects of EHP infection and increase the survivability of *P. vannamei* shrimps by blocking the proinflammatory events triggered via the activation of the NF-κB pathway.

## 4. Discussion

Shrimps infected with *Enterocytozoon hepatopenaei* (EHP) will develop hepatopancreatic microsporidiosis (HPM), an infection known for its impact on the on-farm growth and performance of crustaceans [26]. *Enterocytozoon hepatopenaei* (EHP) is an intracellular pathogen characterised by the transfer of germinated spores into the cytoplasm of the host cell to complete its life cycle and initiate infection [1]. As such, the identification of interventions designed to reduce spore germination and subsequent infection and cell death is vital for the industry [4].

Our results indicate that the natural antimicrobial mixture (Aq) was able to reduce spore germination and afterwards improve cell viability in EHP-infected SGP cells. Spores are also vital in protecting the microsporidia against environmental stress and during adherence and infection of gut cells [27]. Our results indicated that Aq can significantly prevent the attachment of spores to SGP cells; however, the most impact was detected when SGP cells were pretreated prior to infection. These results suggest that a prophylactic application of such interventions can be most effective and this approach was previously demonstrated with other infection models [21,28].

Previously it has been shown that mixtures of natural antimicrobials can reduce and prevent parasitic infections in shrimp resulting in improved survival rates in vivo. The current antimicrobial mixture using (Aq) was earlier shown to reduce *N. messor* virulence by avoiding actin polymerization and improving cell-membrane integrity. The antipathogenic mechanism includes increased SOD and CAT activity, lower H_2_O_2_ levels and inactivation of the ERK signal transduction pathway [14]. Our study shows that, in the case of EHP infection, we have also observed increased CAT and SOD activity; however, the regulatory mechanism involves the inactivation of the NF-κB pathway by Aq, an effect caused by the reduction in H_2_O_2_ production. The involvement of H_2_O_2_ in NF-κB activation was previously described as being mediated through post-translational modifications, specifically serine phosphorylation of p65 [29]. Our study also indicates that Aq can post-translationally modify the activity of NF-κB by removing the Ser phosphorylation of p65. Most of the three major groups of parasites (protozoa, helminths, and ectoparasites) are indeed known for their role in causing a proinflammatory response in infected eukaryotic cells by activating the NF-κB pathway [13]; however, in the case of EHP, this is the first report of such events taking place. Moreover, natural antimicrobials, such as plant extracts, can indeed ameliorate or inhibit reactive oxygen production (ROS) and elevate SOD and CAT levels by decreasing NF-κB activity levels [30]. SOD [31] or CAT [32] are in the first line of defense against excess H_2_O_2_ during infection. Both enzymes play an important role in balancing the levels of H_2_O_2_ produced and natural antimicrobials have been previously identified to modulate their expression [33]. In this current study, we show that following an oxidative burst caused by the EHP spore infection, Aq was able to reduce the levels of H_2_O_2_ produced and significantly upscale the production of SOD and CAT. These results indicate that Aq can reduce the oxidative damage caused by EHP infection and improve the host response by upregulating the antioxidative mechanisms.

Beyond its role as a responder to the infection oxidative stress described above, the NF-κB signalling pathway is also important in regulating the expression of antimicrobial peptides (AMP). In shrimp, WSSV infection activated the NF-κB pathway and triggered the expression of AMPs (lysozyme, crustins, and penaeidins) [20]. Our study shows that in the case of EHP infection, the levels of lysozyme, crustins, and penaeidins are significantly increased in infected shrimp followed by a reduction in the presence of 0.5% Aq. This reduction could be caused by the ability of Aq to block the activation of the NF-κB signalling pathway by EHP.

## 5. Conclusions

**Figure 6:**
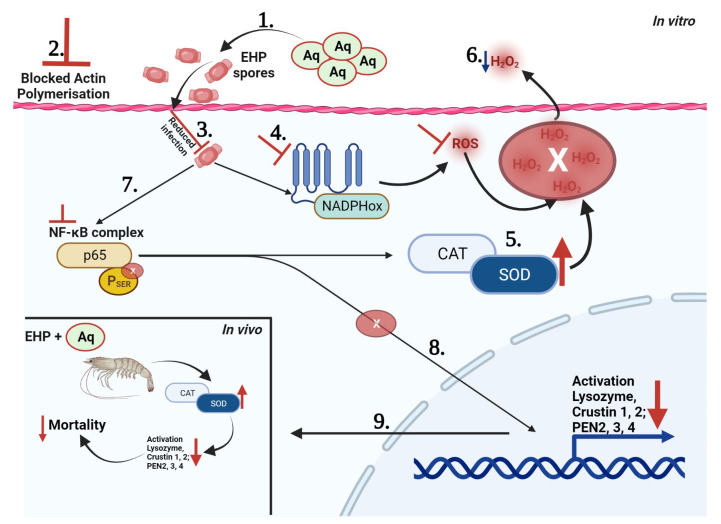
The impact of Aq on EHP infection in vitro and in vivo using a shrimp-gut model of infection.

Herein, we show, for the first time, that mixtures of natural antimicrobials, such as Aq, can prevent EHP spore maturation and reduce infection of shrimp-gut epithelial cells (Figure 6(1)). In vitro, Aq prevented actin polymerisation (Figure 6(2)) and reduced the number of EHP spores able to infect SGP cells (Figure 6(3)). The presence of Aq during the infection of SGP cells prevented the trigger of the NADPH oxidases (Figure 6(4)), increased the expression of CAT and SOD (Figure 6(5)), and reduced the levels of H_2_O_2_ produced and released (Figure 6(6)). The increase in CAT and SOD production was associated with NF-κB dephosphorylation (Figure 6(7)). Moreover, the loss of NF-κB dephosphorylation also led to a decrease in the production of AMPs (lysozyme, Crustin 1 and 2, and PEN2, 3 and 4) (Figure 6(8)). In vivo, a significant decrease in mortality was observed, events which were corelated with the observations made in vitro, suggesting an increase in the production of CAT and SOD (Figure 6).

Using blends of natural antimicrobials improves nutrient utilization in diseased shrimp and are increasing their resistance to bacterial infections with an ultimate positive impact on the survival rates [34]. Our findings are the first to suggest that natural antimicrobials have the potential to reduce the devastating impact of EHP infections in shrimp farming.

## Figures and Tables

**Table 1:** PCR primers used to determine the mRNA levels of PEN 2, PEN 3, PEN 4, lysozyme, crustin 1, and crustin 2.

AMP	Primer Name	Primer Sequence (5′-3′)
*Lysozyme*	Lysozyme-F	AAGACACCGAACGATGGAAG
	Lysozyme-R	TGGGGGACTCGTTCTTTATG
*Crustins*	Crustin1-F	GTCGCAGTGCAGGTACTGGT
	Crustin1-R	TAGTCGTTGGAGCACGTCTG
	Crustin2-F	ATCAGCAGGGGAACAAGAGA
	Crustin2-R	CGGACTCGCAGCAATAGACT
*Penaeidins*	PEN2-F	GCATCAAGTTCGGAAGCTGT
	PEN2-R	ACCCACATCCTTTCCACAAG
	PEN3-F	CTCTGGCTTGTGGAATGGAT
	PEN3-R	GCATGGATTCACTTCCTCGT
	PEN4-F	ATGCTACGGAATTCCCTCCT
	PEN4-R	ATCCTTGCAACGCATAGACC

## Data Availability

Not applicable.
